# Shale Gas Development and Community Distress: Evidence from England

**DOI:** 10.3390/ijerph17145069

**Published:** 2020-07-14

**Authors:** Feizel Aryee, Anna Szolucha, Paul B. Stretesky, Damien Short, Michael A. Long, Liesel A. Ritchie, Duane A. Gill

**Affiliations:** 1 Department of Social Sciences, Northumbria University, Newcastle upon Tyne NE1 8ST, UK; feizel.aryee@northumbria.ac.uk (F.A.); anna.szolucha@northumbria.ac.uk (A.S.); 2 Healthy Living, Department of Social Sciences, Northumbria University, Newcastle upon Tyne NE1 8S, UK; 3 Human Rights Consortium, School of Advanced Study, University of London, London WC1E 7HU, UK; damien.short@sas.ac.uk; 4 Department of Sociology, Oklahoma State University, Stillwater, OK 74078-4062, USA; michael.long@okstate.edu (M.A.L.); liesel.ritchie@okstate.edu (L.A.R.); duane.gill@okstate.edu (D.A.G.)

**Keywords:** shale gas, hydraulic fracturing, fracking, mental health, stress, impact of events scale

## Abstract

This research examines psychosocial stress associated with shale gas development through the narratives of residents and the Revised Impact of Event Scale (IES-R). We carried out our research in three of England’s communities impacted by shale gas development. To gather data, we conducted qualitative interviews and engaged in participant observation in all three communities and conducted a quantitative survey of residents. From our qualitative interviews it was apparent that the residents we spoke with experienced significant levels of stress associated with shale gas development in each community. Importantly, residents reported that stress was not only a reaction to development, but a consequence of interacting with industry and decision makers. Our quantitative findings suggest that a significant portion of residents 14.1% living near the shale gas sites reported high levels of stress (i.e., scoring 24 or more points) even while the mean IES-R score of residents living around the site is relatively low (i.e., 9.6; 95% CI 7.5–11.7). We conclude that the experiences, of the three English communities, reported in the qualitative interviews and quantitative survey are consistent with the reports of stress in the United States for those residents who live in shale gas communities. We therefore suggest that psychosocial stress is an important negative externality, which needs to be taken seriously by local planning officers and local planning committees when considering exploration and development permits for shale gas.

## 1. Introduction

Shale gas development is expanding globally and often relies on unconventional hydraulic fracturing to access gas in the subsurface rocks by injecting water, sand and chemicals into the ground at high pressure. An emerging set of literature suggests that this type of development might have a negative impact on the mental health of residents living near extraction sites [1,2,3]. In particular, recent studies carried out the in the United States (US) suggest that residing near active hydraulic fracturing wells can lead to poor mental health outcomes [4,5,6]. Moreover, in the UK, issue of community stress in shale gas communities, even at the exploration stage, has been raised as a major concern [7,8]. If it can be established that adverse mental health outcomes are associated with shale gas development, then it should undoubtedly be a key consideration in shale gas policy and the planning and permitting process. Few studies explicitly examine stress associated with shale gas development outside of the US. Thus, the state of knowledge is largely based on US scholarship. Importantly, it might be the case that the association between shale gas development and stress are different in the UK than in the US given the unique social demographic (e.g., population density) and geological variations between the two countries. As a result, it is uncertain if, or how, initial US findings regarding shale gas development related stress might be replicated in the UK.

The aim of this research is to (1) examine the narratives of residents living in proposed unconventional gas communities in the UK to begin to understand how they may experience stress and (2) determine if stress brought on by shale gas development can be captured empirically using the Revised Impact of Event Scale (IES-R) [9,10,11]. To meet these two aims, the present investigation employed qualitative and quantitative methods to explore potential distress associated with shale gas development, starting with the proposal to develop shale gas. We conducted ethnographic interviews and participant observation to understand how unconventional gas development creates stress among residents in three UK communities: (1) Preston New Road in Lancashire, where noncommercial unconventional hydraulic fracturing began in 2018 and continued until a moratorium in England prevented it in late 2019; (2) Ellesmere Port in Cheshire where a group of residents worried that a proposed natural gas testing might lead to unconventional hydraulic fracturing; and (3) Kirby Misperton in North Yorkshire, a proposed shale gas site where unconventional hydraulic fracturing equipment was staged and then later removed. We supplemented our qualitative data with results from a quantitative survey of residents living near the unconventional hydraulic fracturing well pad in Preston New Road.

The remainder of this manuscript is organised into four sections. First, we explain the theoretical perspective that guides our examination of shale gas development and stress. Second, we review prior research on mental health, wellbeing, stress and shale gas development. Third, we describe our methods for identifying and measuring stress. In that section we explain how our qualitative approach leads us to believe that shale gas distress can be measured quantitatively, using the Revised Impact of Events Scale [9]. Fourth, we present our qualitative and quantitative results regarding shale gas development and stress. Finally, we conclude by suggesting that shale gas development is stressful because of the future threat it represents and the direct harm it can cause. The findings lead us to propose that local government planners and policy-makers should take into account the harm caused by stress when considering issuing permits to the industry [1].

## 2. Theoretical Perspective

Not all stress is detrimental [12]. However, as discussed below, much of the existing research that focuses on shale gas development suggests that the stress associated with various aspects of that development is damaging. We focus on the harmful impacts of shale gas by drawing upon Folkman and Lazarus’ [13,14,15] modal of transactional stress. In particular, Lazarus and Folkman [15] propose that stress is often the result of an individual’s subjective interpretations of harm, threats or challenges. This transactional view of stress means that each person’s interpretation of an event unfolds over time and shapes their own unique experiences. We suggest that there are, nevertheless, patterns to these subjective interpretations when it comes to shale gas development. Lazarus and Folkman find that when an individual interprets an event as threatening (primary appraisal), they then determine whether they can cope with that stressor (secondary appraisal). In many instances, individuals cannot cope with the threats that they face and therefore suffer from negative stress [14,15]. In other instances, individuals may believe that they can change the situation through direct coping, for example, then they may see a threat as a challenge to overcome (e.g., positive stress).

In applying the Folkman and Lazarus [14] interpretive framework to shale gas development in the UK, we hypothesise that residents living near proposed shale gas development sites will have higher levels of stress. In particular, some members of the community are likely to believe shale gas development will cause harm to their health, the environment and way of life. In the UK, it is largely the prospect, rather than the experience, of shale gas development that leads to distress [14]. Importantly, this is only partially similar to the situation in the US where shale gas development is not only a result of perceptions of harm to come, but critically, a result of direct harm and loss produced by the extraction process itself [3,5,16,17,18]. That is, the US has engaged in shale gas development for years and the impacts of that development are increasingly being evaluated and found to have a variety of negative impacts on the community. In the UK, shale gas development remains in its infancy, and there are few studies of the direct impacts of shale gas development. Nevertheless, initial qualitative research in the UK suggests that residents who oppose development by engaging in local democratic behaviour may be directly impacted when challenging the industry, local planners and regulators. That is, community members that oppose shale gas development, and especially unconventional hydraulic fracturing, report that engaging in public speaking, testifying at appeals, fundraising and protesting take a toll on their emotional wellbeing even when they see unwanted development as a potential challenge to be overcome [14].

Prior to determining how residents describe their experiences with shale gas development, we briefly summarize the existing literature in this area. We do this by summarising qualitative and quantitative studies of stress and shale gas development. Importantly, as previously noted, while many US studies attribute psychosocial stress to the actual processes associated with hydraulic fracturing, we suggest that stress is also an outcome of perceptions of harm shale gas development will cause, even before the process of extraction begins.

## 3. Literature Review

Living near shale gas development can be stressful [16]. Ferrar et al. [17] found that stress was one of the most frequent outcomes described by residents living near active hydraulic fracturing sites. Research also documents that adverse mental health outcomes are related to both the distance individuals reside from hydraulic fracturing wells and the size of those wells [5]. Importantly, the announcement of shale gas development within a community can be viewed differently by residents. The existing literature suggests there are four different ways people residing near shale gas development activities perceive it as threatening and distressful. First, residents are often concerned about their health. Second, residents worry about physical impacts associated with unconventional hydraulic fracturing such as light, noise and induced earthquakes. Third, residents may be anxious about changes to their way of life that harm the social and natural environment. Finally, residents who attempt to resist or support shale gas development can also experience high levels of distress when interacting with industry, regulators, planners and government concerning developments in their communities.

### 3.1. Threats to Health

Hydraulic fracturing that accompanies shale gas development is stressful because it is perceived to threaten physical health [4,19,20]. Brasier et al. [2] (p. 38) learned that health experts in Pennsylvania were concerned about hydraulic fracturing and shale gas development, saying “we’ve done a number of assessments…there are some community perceptions that there are some health issues.” Fisher et al. [19] also uncovered that residents in two Ohio (USA) counties that lived within 5 miles of hydraulic fracturing well faced considerable levels of stress due to their health concerns about those wells. The researchers found that physical health was a core category of concern and many residents were anxious about air pollution and water contamination. Fisher et al. also discovered that some residents thought that hydraulic fracturing was responsible for headaches, respiratory symptoms and skin conditions. Importantly, distressed residents told the researchers about cancer clusters that they believed were attributable to nearby hydraulic fracturing activity. Finally, local workers in the hydraulic fracturing industry pointed out that they experienced chemical burns, a loss of consciousness from noxious chemicals and musculoskeletal injuries from heavy on-the-job lifting [19].

Leaking wells are also a threat to health. For instance, Perry [20] found that hydraulic fracturing sites in Bradford County (Pennsylvania) caused residents to worry that they would become ill from leaking wells. Perry (p. 88) describes a situation where a local landowner with no history of mental illness was diagnosed by a clinical psychologist as having a “severe stress reaction.” The landowner believed that chemical leaks were threatening his home and the health of children in the community. Mayer et al. [6] also document that residents in shale gas communities in Colorado (USA) were distressed because they were anxious about the impact of hydraulic fracturing on their respiratory health. Finally, Casey et al. [5] suggest that among a sample of mothers who were not anxious or depressed at the time of conception, those who lived closer to shale gas development activity had elevated levels of anxiety, perhaps because of worry for the physical health of their children.

### 3.2. Threats to Physical Environment

Residents living near shale gas development activities may have higher levels of stress as a result of environmental impacts such as noise, light and seismic activity. Evensen and Stedman [21] found that residents who held negative views about the shale gas industry were more likely to complain about air pollution, truck traffic and general noise. Drummond and Grubert [22] who conducted interviews with residents living near earthquakes in Oklahoma, found that one of the most obvious narratives among a sample of participants was centred on the uncertainty of seismic activity. Many of the participants they spoke with suggested that the uncertainty surrounding seismic activity that resulted from a lack of information contributed to significant levels of stress and emotional damage. Korfmacher et al. [18], for example, suggests that light produced by hydraulic fracturing operations contributed to stress among nearby residents. Perry [1] also states that associated industrial traffic is often mentioned as stressful by residents living near shale gas wells. Finally, Fisher et al. [19] (p. 94) note that as trucks and machinery were constantly operating (e.g. “There [was] constant dozer work, constant noise all the time”) and that the noise from those activities became a source of stress to nearby residents. The researchers proposed that the truck-related sounds, which continued after drilling stopped, prevented people from falling asleep and they were awakened sporadically, contributing to their self-reported levels of stress.

### 3.3. Threats to Community

Residents in shale gas communities may feel stressed because they believe their community’s natural, social and economic resources are being threatened. For instance, Perry [1] (p. 82) reports that residents believed that the arrival of the shale gas industry had forever altered the connections they had with their “family histories, childhood memories, their lands, their neighbours and communities, the past, and the present”. Brasier et al. [2] (p. 44) also find that many residents feel like the overall negative community impacts of shale gas development are simply “dropped to the wayside because it’s so stressful.” Finally, Lai et al. [23] finds that threats to social and ecological systems in a community can lead to significant levels of psychological stress among residents living near unconventional hydraulic fracturing sites.

While most studies suggest that residents living near hydraulic fracturing sites felt that their communities were threatened, Drummond and Grubert [22] discover that community attachment prevented residents from speaking openly about their concerns and therefore suffer from greater levels of stress. That is, some of the participants the researchers talked with in Oklahoma said that while they worried about their own property, they did not want to speak out against economic development that others believed helped the community (e.g., “I’ve got probably ten different people in town that said to me, yeah, my house is damaged, but they won’t say it in public”) [22] (p. 132). Notably, the idea that shale gas extraction can help communities has also been the subject of study. For instance, Evensen and Stedman [21] find that some of the residents in the six US and Canadian communities they studied welcomed shale gas development as it improved wellbeing and community survival by encouraging important economic development.

### 3.4. Stress from Active Involvement

Stress from involvement in support or opposition to shale gas development also impacted communities directly. Residents are likely to report levels of stress as a result of their interactions with various agencies, public speaking, taking part in appeals, fundraising and protesting. While these events could be viewed as encouraging positive reactions to a threat, not all residents viewed it this way. Specifically, opposition and support of shale gas development was often described as highly stressful [24]. This is also consistent with some of the protest literature that documents higher levels of stress as a result of engaging in political activism [25,26]. Likewise, previous research on shale gas in the UK suggests that some residents discover that participating in the democratic process is unlikely to change shale gas development in their community, leading them to describe feelings of unwanted distress rather than empowerment [7].

## 4. Materials and Methods

**Figure 1:**
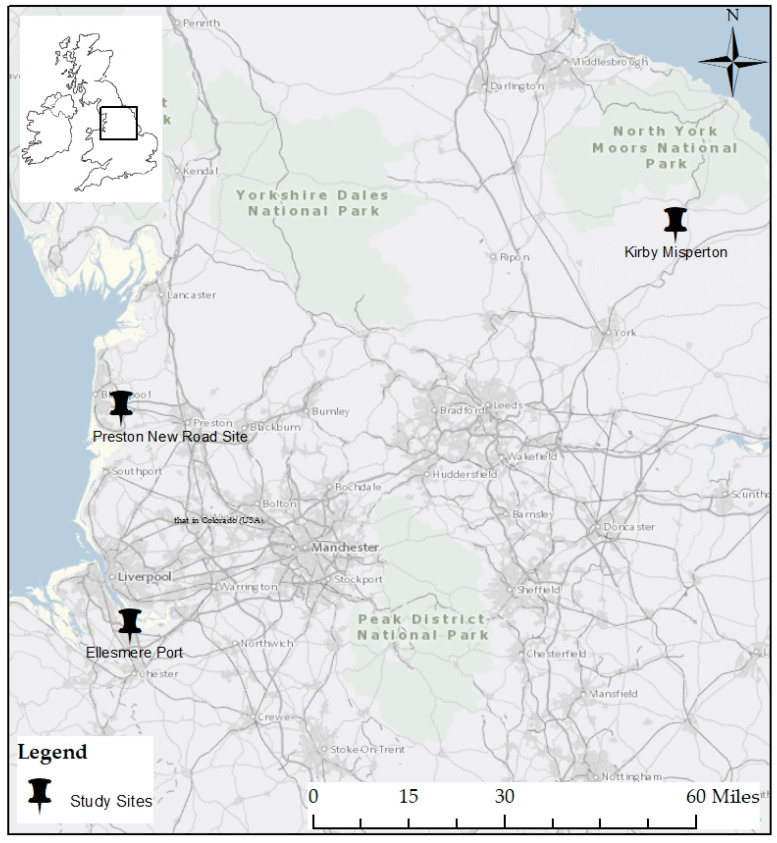
Map of study locations.

To examine the concept of residential stress in a shale gas community, we studied three UK communities. Two of those communities (Ellesmere Port in Cheshire and Kirby Misperton in North Yorkshire) faced proposals for natural gas development that have not yet been successful. The development in North Yorkshire targeted shale gas, while there was substantial controversy over the specific geological target and character (conventional/unconventional) of the gas development in Cheshire. One of these communities, Kirby Misperton, faced significant levels of protest as a result of the proposal to build a hydraulic fracturing well pad within walking distance of the village [27]. The third community, Preston New Road in Lancashire, was home to an active hydraulic fracturing well pad that operated in 2018 and 2019 prior to being halted when England’s hydraulic fracturing moratorium was put in place by the central government [28]. The locations of these three communities are displayed in Figure 1.

### 4.1. Qualitative Interviews and Participant Observation

To investigate how residents and other stakeholders describe their experiences with shale gas development, we used participant observation and conducted qualitative semi-structured interviews with over 100 participants across the three locations in Figure 1. Observations were carried out at local planning authority meetings, public inquiry hearings, local grassroots events, protests and the events of national regulatory agencies. One researcher (Szolucha) made multiple visits to all three sites between 2015 and 2020 and lived within a short distance of the actual hydraulic fracturing site in Lancashire for 15 months and the potential site in North Yorkshire for 6 months.

High-visibility stakeholders, such as community and local government leaders, were identified using publicly available information. Less visible stakeholders, such as those involved in the hydraulic fracturing supply chain, farmers, business owners, protesters, police officers and other residents, were identified through snowball and purposeful sampling as well as residential canvassing. The sampling sought to gain a good representation across different ages, genders and class positions. Interviews were continued until saturation was reached in Lancashire and North Yorkshire. All interviews were transcribed verbatim while extensive field notes were taken during participant observation.

The aim of participant observation and interviews was to recreate the social processes behind the construction of meaning in the case of shale gas development. It was in this capacity that we observed that stress and anxiety emerged as a recurring theme from the very onset of our work [7,29]. As that research shows, stress related to hydraulic fracturing consumes people’s lives, leads them to engage in avoidance behaviours and creates irritability.

### 4.2. Quantitative Survey

**Figure 2:**
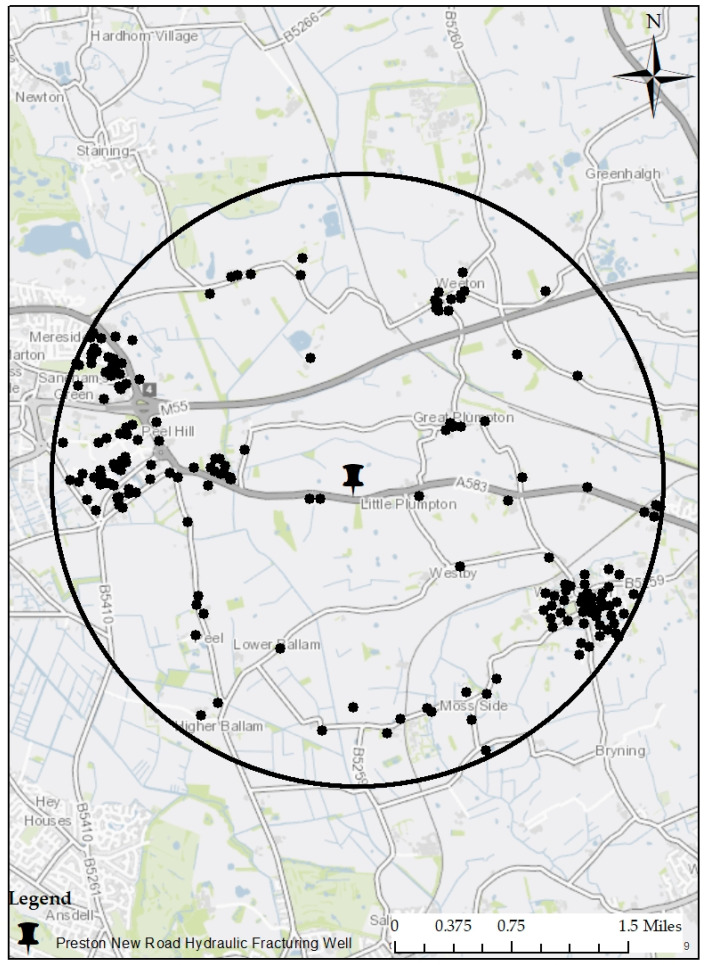
Post code areas surveyed near Preston New Road.

To build on the emerging qualitative results (see Short and Szolucha [7]) and get a quantitative estimate of the proportion of residents that may be experiencing stress related to hydraulic fracturing, we used the Revised Impact of Event Scale (see Appendix A) in a survey-based instrument administered to Preston New Road residents living within two miles (see Figure 2) of the hydraulic fracturing sites at Preston New Road in 2017 (i.e., before hydraulic fracturing began). As Figure 2 indicates, residents were surveyed at dozens of locations surrounding the proposed hydraulic fracturing site.

The questionnaire was mailed in a self-addressed envelope to a set of 1136 addresses in the area displayed in Figure 2. A total of 198 household residents responded to the survey, making the response rate 19%. A comparison of the sample to the population is provided in Appendix B where population data was derived from the Office of National Statistics in the case of (1) demographic data for the 13 Output Areas surrounding Preston New Road and (2) income data for the entire Fylde Council. As those comparisons suggest, the sample is fairly representative of the surrounding population in the case of weekly income (sample estimate £600–£1249 vs. population value £614); the percentage of White British (99.5% sample vs. 97.8% population) and the percentage of females (59.9% in the sample vs. 51.5% in the population). The sample, however, did have higher rates of university educated respondents (51.7% of the sample had a university degree compared to 32.2% of the population); lower levels of full time employment (55.4% of the sample was employed full time compared to 70.9% of the population) and a higher median age (between 55 and 64 years old in the sample compared to 48.6 years old in the population). In short, it appears that the sample is slightly more educated, a little older and less likely to be working full time. There is no reason to believe that small variations between the sample and population had any major impact on the reported levels of stress we estimate.

Following up on themes in the qualitative research we suggest that distress from hydraulic fracturing can be measured using the IES-R. The original IES was developed by Horowitz, Wilner and Alvarez [10] and uses 15 questions to measure distress resulting from an event in two main categories, intrusive thoughts and avoidance behaviours, while the revised version has 22 questions and includes hyperarousal [9]. Importantly, while we may be the first researchers to use the IES-R to study shale gas development, we are not the first researchers to use the IES or IES-R to study threats from the natural environment. For instance, Grainger et al. [30] used the IES to track levels of distress among patients receiving Eye Movement Desensitization and Reprocessing treatment (compared to a control group) after Hurricane Andrew. Likewise, Heir et al. [31] found that post-traumatic stress, as measured by the IES, did not completely subside among three groups of Scandinavian tourists who were exposed to a tsunami while on holiday. Researchers have also used the IES to examine victims of floods and earthquakes [32,33,34]. The IES or IES-R has even been used to examine stress associated with levels of pollution in general. For example, Ho et al. [35] studied a population of Indonesia residents exposed to haze from fires in neighbouring countries and found that the average IES-R score was 18.5 (Std. Dev. = 11.7), which they classified as mild psychosocial stress.

To get information from residents living near Preston New Road, each participant was read the following statement:

Below is a list of difficulties people sometimes have after stressful life events. Please read each item, and then indicate how distressing each difficulty has been for you during the past seven days with respect to hydraulic fracturing at Preston New Road. How much were you distressed or bothered by these difficulties?

At the conclusion of this statement each participant was asked to respond to a list of items scored from 0 (“not at all”) to 4 (“extremely”) that are added together to compute a total score ranging from 0 (no stress) to 88 (severe distress) as well as subscale scores for the concepts of intrusion, avoidance and hyperarousal. Intrusion measures those thoughts that cause people to think about or re-experience traumatic events, such as nightmares and imagery of the event, and scores on the subscale range from 0 to 32. Avoidance measures the averting of situations, ideas and feelings that bring back thoughts of the event and scores on the subscale range from 0 to 32. Hyperarousal measures things associated with high stress situations and ranges from 0 to 24.

## 5. Results

### 5.1. Qualitative Findings

From the start of our ethnographic research into the social impacts of shale gas exploration in the UK, we found that the prospect of shale gas development, even before preparation began, had a significant effect on local residents. Participants in the three communities reported feelings of stress and anxiety caused by their concerns about their health and the impact of shale gas development on the community, including the natural environment. For example, during a planning inquiry in 2016, a local resident who lived in the vicinity of the proposed Preston New Road site summarised it thusly, struggling to hold back tears:

Two estate agents stated, should fracking go ahead, our property would be potentially worthless... It has also affected properties selling in Lytham St Annes belonging to my recently deceased parents… in the last two years, we’ve lost nana, mum, dad and our beloved dog. My younger brother nearly died and only after emergency triple heart bypass did he recover. Last year, I donated a kidney to him. I can honestly say that organ donation was far less stressful than the constant worry for my family, friends and community for the last 2 years. In addition to this, one of my sons has a genetic disorder... and already struggles to breathe. We are 800 m from the potential fracking site and now face the prospect of living downwind of flaring methane for 90 days at a time. For me, this is a massive and legitimate concern. There is no offer of compensation from either the government or the shale gas industry and no baseline health monitoring has been implemented. [Shale gas operator] is also opposing any health monitoring. Who will take responsibility for our present and future health conditions potentially resulting from shale gas development? Where is our protection? We are in a position where we cannot afford to move but we simply cannot stay. Finally, I work part-time for a friend in a small convenience store not far from here. I would like to make it known that on Thursday, 4th of February two gentlemen offered to pay myself and a friend 50 pounds each to attend Blackpool Football Club [where the planning inquiry was] on Tuesday the 9th of February for one hour to back fracking… [She breaks down and cries.] Our communities have suffered enough and [the company] do not and will never have social licence to frack the Fylde.

The feelings of distress reported in Lancashire were also experienced by local residents in Cheshire and North Yorkshire. Follow-up interviews showed that the stress and anxiety intensified as shale gas activities progressed. The stress and anxiety also seem to have a long-lasting impact on residents, farmers, business owners and other stakeholders who were not actively involved in either supporting or opposing hydraulic fracturing. In the following subsections, we present some of our observations of the most common ways in which distress manifested itself across the three locations in our study, namely, through expressions of intrusion, avoidance and hyperarousal.

#### 5.1.1. Intrusion

At the beginning of our research, residents who perceived shale gas development as a threat reported experiencing feelings of anxiety that were intruding on their daily lives. One resident who lived in close proximity to the Preston New Road hydraulic fracturing well pad, described shale gas development as a “dark, dirty cloud” that was hanging over him and his family:

My greatest aspiration is for life to resume a sense of normality and for this threat to go away and for us to think to the future, to the bright happy days. But this is like a dark, dirty cloud that is hanging over. It’s like this neighbouring country—that has different beliefs and ideals – is going to invade here. It’s that same oppressive effect that I feel and a lot of other people feel as well. Because it’s taking away from what we work for—for our future and for our children’s’ future.

Those local residents who were actively involved in the planning process, and/or with protests, additionally found that their engagement was “taking over” their lives, since what at first might have been described as positive stress in the democratic process was transformed into a life of struggle. As one local resident from Lancashire noted in 2015–2016:

It’s the lack of balance in people’s lives anymore… for people to have a decent quality of life, you’ve obviously got your home, your work, your family, you have those pillars… that is not the life that any of us are living. We’re sole-track, totally going down this route and there is nothing else.

Another resident from Lancashire suggested:

It has infiltrated people’s lives to such a degree that they basically almost live and breathe fracking… You look for a bit of escapism where you switch off for a while, but there is always something that lurks in the background and raises its head again…

In follow-up interviews with residents, we found that the levels of stress they were experiencing were not decreasing, but increasing as shale gas development progressed:

I think since we last spoke it’s probably got even more stressful because since we last spoke we obviously had the public inquiry and that was a tremendous amount of pressure because we were Rule 6 party on the inquiry so we obviously had to engage expert witnesses and the barrister, we had to prepare documentation and the amount of documentation that was required was enormous so it was about getting that printed, getting it all into files because it all had to be bound… I ended up with 30 odd arch lever files of documents and then they all had to be packed up and sent away and they had to get to the inspector by a certain date so that was really stressful.

Throughout our research, some of those who were actively engaged in supporting or opposing shale gas development stopped devoting energy to their cause—reporting that the impact it had on their health was excessive. Still, several residents said that they could not withdraw from their activism because they felt too strongly about their views, as this local opponent from Cheshire explained:

I keep thinking, it’s too much, it’s too much stress on me, too much stress on my husband and my children because it takes up too much of my time and I could be doing other things but I think that once you know about it, it’s difficult to let it go because it just needs to be banned in the UK.

Those participants who decided to remain actively involved in supporting or opposing fracking sometimes struggled with the effects that shale gas development was having on their daily lives, in some cases causing sleep disturbance and other health impacts such as exhaustion from constant engagement in opposition. As this resident from Ellesmere Port in Cheshire reported when asked if they had problems sleeping:

I do because of my anxiety. It gives me anxiety that I might have to do more protesting and I’m exhausted from it. I’m just in Ellesmere Port and I’ve been to Preston New Road, I was in Duttons Lane at Upton all the time and I’ve been doing it for years now and mine isn’t because I like staying at the camps because I didn’t. I came home so it’s exhausting and I don’t think that I can go through that exhaustion again and it does give me sleepless nights worrying about it because I know that I will have to, I’ve got to, people have got to because that’s what’s happened. A lot people have [become] just so disheartened with it, so exhausted through it that they just dropped out or dropped dead as [name]. It’s been a few of them over the years. Standing on that road is not good for health.

In short, a number of residents in the communities we studied suggested that shale gas development enveloped and took over their lives, intruding on their thoughts in a way that they were unable to stop thinking about shale gas development in their community. In some cases, as we have noted, thoughts of shale gas development kept them awake at night.

#### 5.1.2. Avoidance

Avoidance was another way that residents dealt with potential shale gas development in their communities. Unlike intrusion, avoidance was particularly difficult to capture in interviews and is likely underreported. This was made clear when researchers inquired about an interview of a farmer and his family to the famer’s neighbour. The neighbour explained to the researchers that the farmer was so impacted by the conflict in the community that it was simply better to just “let it be” rather than seek an interview with the farmer. Other residents, especially those who support gas exploration, were likely to deny that they find gas development stressful. However, independent observations found high levels of anger, and potentially stress, were displayed in public forums or sometimes in response to requests for an interview. Throughout our research, we interacted with residents, farmers and business owners who responded in an unusually angrily manner to our requests for an interview. Often these were identified as potentially most impacted by shale gas developments because they lived close to hydraulic fracturing well pads. They would hastily deny shale gas was having any effect on their lives, where our ethnographic research would suggest otherwise, or angrily express their frustration with the issue of hydraulic fracturing and assert that they “did not want to have anything to do with it” Tellingly, even those farmers who emphasised that they were not stressed by hydraulic fracturing, concluded when asked whether they would recommend shale gas development to other farmers and landowners:

I could recommend it but it all depends what stress it might give the farmers that do it. Different areas could be different. There might be different oppositions and everything. I don’t think I’d have liked it if it were like the other site at Roseacre [another proposed site in Lancashire], falling out with your neighbours and that wouldn’t have done but there’s nobody, we often joke about it, but nobody is falling out.

Importantly, avoidance remained a strategy that communities used to deal with distress long after the immediate prospect of hydraulic fracturing had passed. Residents avoided talking about shale gas because it was perceived as too controversial, like these residents from Kirby Misperton in North Yorkshire suggested in 2020, two years after the hydraulic fracturing operator removed their equipment from the site:

It really isn’t something that you talk to somebody in the village…we just don’t talk about it because it was just too heated a discussion. It was all sort of we know what’s going on but let’s talk about something else because it was quite consuming for everybody…I think we all sort of just ploughed on.

Avoidance can affect health because people may change their habits and use various substances to cope with stress, like in this account of a resident in Lancashire who was describing the impact that stress of being actively involved in opposing hydraulic fracturing was having on their life:

Previously, I wouldn’t start to drink midweek. I’d just have Friday, Saturday, maybe a glass on Sunday but that would be it because it was always well it was school night or work night or whatever. There is no nights off now really because every night there is just, I mean when you’ve been to a [Gas Company] meeting, and you’ve been that height. I’m a smoker, and last year I ended up hospitalised with lung problems and I’ve literally in the last 2 years gone from, I’ve always smoked about 20 a day, gone up to 30 plus because there are days when I can just eat them. And I put that down to stress. And I’m saying just [Gas Company], a lot of it, the last 6 months has been [Gas Company], the fracking, the whole thing.

#### 5.1.3. Hyperarousal

Hyperarousal has been reported especially by residents who have been actively involved in supporting or opposing shale gas development. It usually manifests itself as constant pressure to research, write and organise, as this anti-fracking local resident from Lancashire reported:

I’m tired and I get a lot more headaches because I’m on a computer a lot more and have to write a lot more. My biggest problem is sleeping, lack of sleep because what I do is work late, wake up in the early hours, think of something I need to do, put it in my mobile, then I look at Facebook or my mailbox. That’s probably why I’m tired. I have been up at 4 am writing reports and sending stuff in the middle of the night because I thought that I needed to have that done. I have not been doing the exercise that I should be doing. I have type 2 diabetes and overweight so I’m not looking after myself properly. I should be eating more carefully but because I’m rushing around, anxious, [I don’t]. I haven’t had to go to the doctors yet but there have been people here who have.

Pro-fracking residents who got involved in supporting shale gas development reported similar experiences concerning their workloads during the height of their campaigns, as this person from North Yorkshire described:

It takes a lot of time. It has taken over my life. I work more than any 40 hour a week on this, particularly at the height of it all. I don’t like being beaten. I like fairness and I like the truth. And when those are not happening, I will fight against it, what they are doing.

Overall, our findings suggest that there was a sizeable group of local residents who experienced stress as a result of planned or actual gas developments in their area. The levels of stress for some of those residents were very high, as some even mentioned post-traumatic stress disorder to describe the impacts hydraulic fracturing has had on their lives, as in the account of this resident from Lancashire:

My life will never be the same again. I do believe we will beat this and I do believe they will go away… I will see them go but think the effect that it will have after they’re gone will be the worst because I think we will all in some way suffer some sort of post-traumatic stress because we’ve lived with this and we’ve been physically and mentally hurt by this and it has affected our lives in such a way that we will never ever again have [a normal life]. We can never go back to what we had 5 years ago or 10 years ago and I think that some people will have a problem adjusting because it’s impacted them very much. The effects and it’s not just us, you have to remember that there’s people like us and communities all over the country who are fighting this. There are going to be affected just like us.

### 5.2. Quantitative Findings

**Figure 3:**
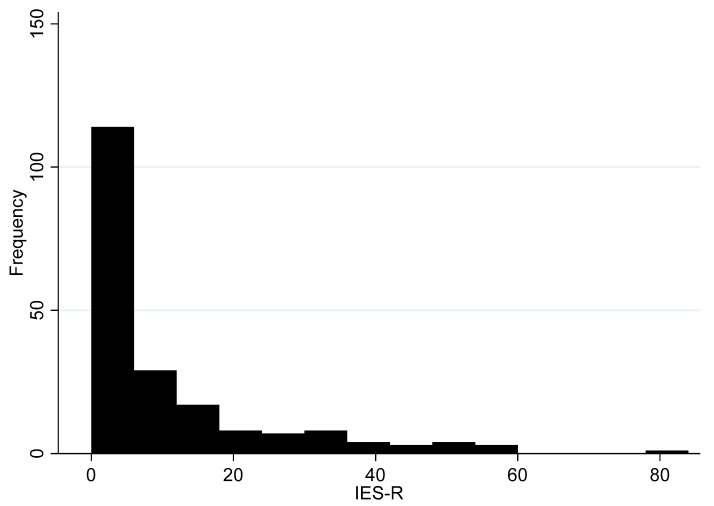
Histogram of Impact of Event Scale-Revised scores (*n* = 198).

We begin our examination of overall IES-R scores in Figure 3. As noted, in that figure, the distribution of overall IES-R scores in the sample of residents’ range from 0 to 84 (on the 88-point scale). Importantly, IES-R appears consistent among the 22 items (α = 0.95), with all items contributing to the scale equally well. This is consistent with previous research on the consistency of the IES-R [36]. The overall mean IES-R score is 9.6 (95% CI 7.5–11.7), which is about half of the Ho et al. [35] estimate of 18.5. Thus, this does not appear to be a substantively large average score but we have to bear in mind that this survey was carried out after the construction of the site began, but before any hydraulic fracturing started. A follow-up survey is in preparation. Moreover, we estimate that 40.9% of all residents scored “0”, indicating that they “never” experience shale-gas-related stress. While research on IES-R cut-off scores are generally vague, some studies suggest that scores of 24 or greater are indicative of PTSD [37]; scores of 33 or more suggest PTSD is probable [38]; and, scores of 37 or more are indicative of PTSD and likely to cause levels of stress that are high enough to supress the functioning of the immune system [39].

Overall, the distribution of IES-R scores suggests that 14.1% of the residents who answered the survey scored 24 or more on the IES-R; 8.1% scored 33 points or more and 7.1% scored 37 or more points. Thus, while the overall IES-R score is low (as nearly 40% of residents surveyed in the community are not stressed), there is still a sizable portion of residents that are suffering from significant levels of distress that may be indicative of PTSD.

**Figure 4:**
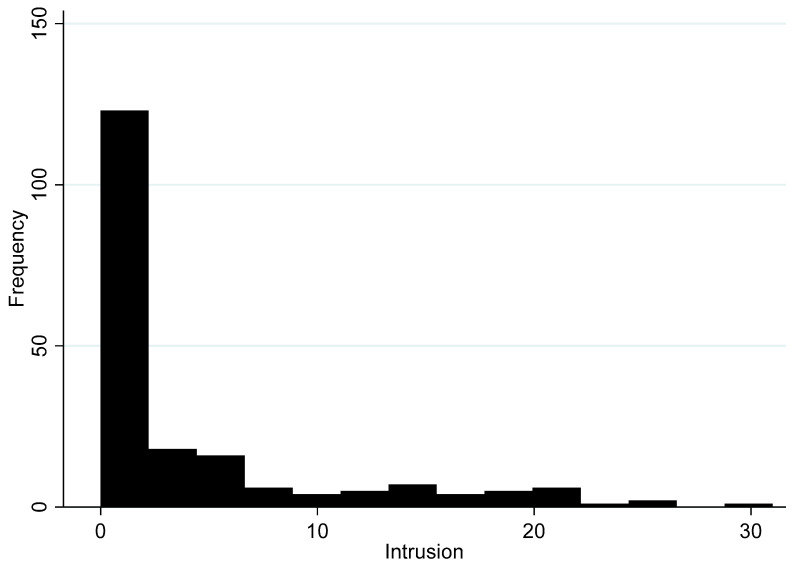
Histogram of intrusion subscale (*n* = 198).

Next, we turn to the IES-R subscores. Recall that the intrusion subscale measures those thoughts and feelings that cause people to think about or re-experience traumatic events such as nightmares and imagery and ranges from 0 to 31 (on the 32-point subscale). We examine the distribution of intrusion scores in Figure 4. First, the intrusion subscale is consistent among the eight measures of the IES-R subscale (α = 0.93).

The average score on the intrusion subscale is 4.2 (95% CI 3.3–5.1) out of a maximum of 32 points. Most residents (50.5%) report that they did not experience feelings of intrusion as a result of shale gas development and were scored “0” on the subscale. The maximum score on the subscale was 31 and represents extreme intrusion. Overall, then, the histogram of intrusion is similar to the overall levels of stress reported in Figure 2, except that a greater percentage of people score “0” on the intrusion subscore than on the overall IES-R (50.5% vs. 40.9%).

**Figure 5:**
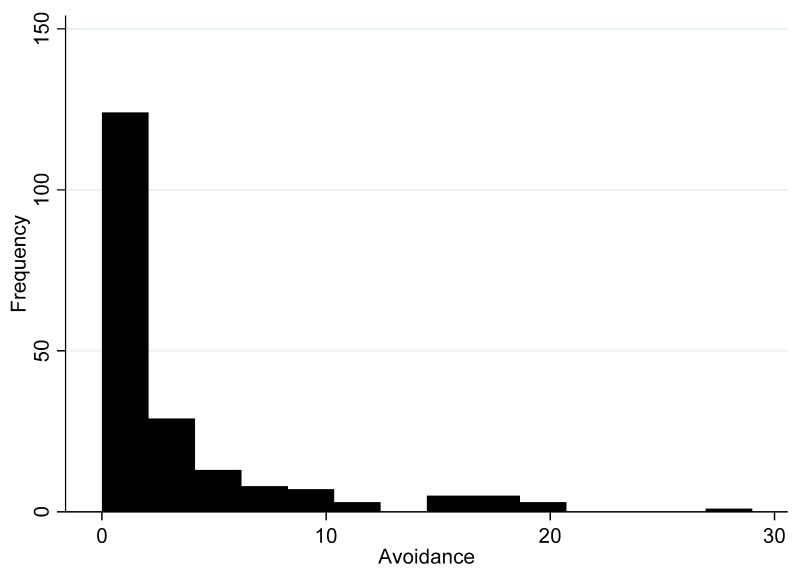
Histogram of avoidance subscale (*n* = 198).

Figure 5 presents the results for the avoidance subscale, which measures the level at which residents report trying to avert thinking about the traumatic situation and the distress it may cause.

Again, the subscale is consistent among the eight items, with an α = 0.88. The maximum score on the subscale was 29 points and a mean score of 3.2 (95% CI 2.5–3.9). Most residents (50.0%) report that they did not engage in avoidance activities and scored “0” on the avoidance subscale. Again, the distribution on the avoidance subscale looks like previous distributions of overall stress and intrusion.

**Figure 6:**
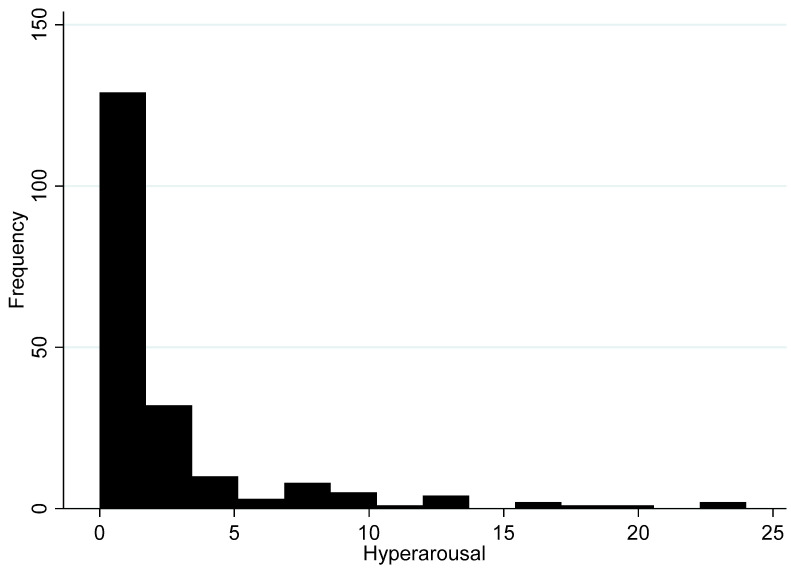
Histogram of hyperarousal subscale (*n* = 198).

Finally, we present the histogram for the hyperarousal subscale in Figure 6. Hyperarousal measures high stress situation behaviours and is composed of six items. The internal consistency among the items is α = 0.87 with a mean score of 2.2 (95% 1.6–2.8) and a maximum score of 24. Overall, 52.5% of all residents living in the shale gas community suggest that they did not experience hyperarousal which is also consistent with the overall IES-R subscale. The distribution of hyperarousal looks much like the distribution of avoidance and intrusion.

Taken together, the quantitative findings support the qualitative results and suggest that the IES-R averages are not particularly high within the overall Preston New Road community. However, there is a sizeable group of residents that are likely to be suffering from severe stress because of shale gas development in their community, even before hydraulic fracturing begins. Importantly, no single type of stress stands out in the qualitative or quantitative literature as particularly relevant. That is, all forms of stress are distributed relatively equally according to the frequency distributions in Figure 3, Figure 4, Figure 5 and Figure 6. We discuss these findings further below and offer our conclusions.

## 6. Discussion

An increasing number of studies find that residents living near unconventional hydraulic fracturing sites suffer from poor mental health, including elevated levels of stress [2,4,6,16,17,19]. Nevertheless, additional research is needed, especially in countries other than the United States where findings may not be replicated. As a result, the purpose of this research is to contribute to the shale gas development literature by examining distress in the UK. We do this by examining qualitative and quantitative data collected in two communities where residents believed shale gas would be developed and one community where unconventional hydraulic fracturing was occurring. Our qualitative findings suggest that shale gas development has had significant impacts on residents in each community. This stress tended to focus on intrusive and avoidance activities as well as hyperarousal. Moreover, many residents were emotional in recounting their experiences with development.

Our quantitative findings extend our qualitative work and suggest that stress may be captured empirically using the IES-R. We discovered that many residents see shale gas development as a threatening event, especially because it often uses unconventional hydraulic fracturing. Overall, it is important to point out that our quantitative findings suggest that a significant portion of residents living in the communities we studied are distressed because they view shale gas development as a future threat. The distribution of IES-R scores suggests that 14.1% of the residents who answered the survey scored 24 or more on the IES-R; 8.1% scored 33 points or more and 7.1% scored 37 or more points. These findings are not out of line with US estimates of community stress among those residents who live near operational and market producing shale gas wells. For instance, Mayer et al. discovered that between 8.9% and 17.3% of residents in the communities they studied “agreed or strongly agreed” that shale gas development increased their levels of stress [6].

While we find that a high percentage of residents are distressed as a result of shale gas development the average level of stress is, nevertheless, relatively low. In particular mean IES-R scores are found to be 18.5 in the case of serious air pollution [35] and 9.6 (95% CI 7.5–11.7) for shale gas development in our sample at Preston New Road. Thus, in our data at least, mean stress levels are about half the size of the stress levels reported for a significant environmental hazard. However, importantly, our survey was carried out before any hydraulic fracturing took place, and our follow-up research will investigate whether the levels of stress changed after locals experienced the impacts of fracking such as multiple earth tremors. Relatively speaking, these findings again underscore the impact of shale gas development, even at the planning stage. That is, residents in the UK perceive the time when shale gas development is being planned as a stressful event that is a harbinger for harm to themselves and their community. Thus, we might hypothesize that community stress will therefore increase as shale gas development proceeds.

In terms of further research, we suggest that more needs to be done to empirically measure levels of community stress around shale gas development sites at different stages of development to learn more about the ways in which stress may vary over time. In particular, the IES-R appears to be compatible with our qualitative findings and could be used to make comparisons across communities and over time. Moreover, it is not yet clear in any research how long the impacts of shale gas development are likely to last. Research on traumatic events, such as environmental disasters, suggests that levels of stress may remain high for many years [40]. We therefore ask if the same is true of shale gas development?

## 7. Conclusions

When it comes to social policy, policymakers, planners and local councillors should, at the very least, seriously consider the impact of stress on communities, starting as early as the planning phase of shale gas development, and add it to the impact balance sheet when making planning decisions. In particular, as soon as a shale gas exploration application is made, we suggest that, policymakers and planning authorities should ensure that proper plans are in place to detect and mitigate problems related to stress and provide services to ameliorate stress where needed. A more cautionary approach to these findings would, however, support the decision to place a moratorium on hydraulic fracturing for shale gas in the UK given the potential health impacts this research has uncovered.

## Appendix A. Categories of Items in the Impact of Event Scale—Revised

Intrusion Subscale (8 Items)

1. Any reminder brought back feelings about it.
2. I had trouble staying asleep.
3. Other things kept making me think about it.
4. I thought about it when I didn’t mean to.
5. Pictures about it popped into my mind.
6. I found myself acting or feeling like I was back at that time.
7. I had waves of strong feelings about it.
8. I had dreams about it.

Avoidance Subscale (8 Items)

1. I avoided letting myself get upset when I thought about it or was reminded of it.
2. I felt as if it hadn’t happened or wasn’t real.
3. I stayed away from reminders of it.
4. I tried not to think about it.
5. I was aware that I still had a lot of feelings about it, but I didn’t deal with them.
6. My feelings about it were kind of numb.
7. I tried to remove it from my memory.
8. I tried not to talk about it.

Hyperarousal Subscale (6 Items)

1. I felt irritable and angry.
2. I was jumpy and easily startled.
3. I had trouble falling asleep.
4. I had trouble concentrating.
5. Reminders of it caused me to have physical reactions, such as sweating, trouble breathing, nausea, or a pounding heart.
6. I felt watchful and on-guard.

## Appendix B

**Table A1:** Selected demographic characteristics of Preston New Road sample and population.

	Sample	Population (a)
% Female	59.9	51.5
% University Degree	51.7	32.2
% Employed Full Time	55.4	70.9
% White British	99.5	97.8
Median Age	55–64	48.6
Weekly Income	£600–1249	£614 (b)
Weekly Income	£600–1249	£614 (b)

(a) Source: Office for National Statistics, 2011 Census Data for Output Areas. The following output areas were included in Areas calculations: E00126929; E00126951; E00126952; E00126986; E00126987; E00126988; E00126989; E00126990; E00127032; E00127046; E00127047; E00127048; E00127049. (b) Source: Office of National Statistics, Annual Survey of Household Earnings-Resident Analysis Dataset: Weekly income data are for Flyde in 2018.
